# Postnatal depression in British mothers of African and Caribbean origin: a randomised controlled trial of learning through play plus culturally adapted cognitive behaviour therapy compared with psychoeducation

**DOI:** 10.3389/fpsyt.2024.1383990

**Published:** 2024-03-28

**Authors:** Dung Ezekiel Jidong, Tarela Juliet Ike, Maisha Murshed, Christopher Francis, Shadrack Bitrus Mwankon, John Ezekiel Jidong, Juliet Yop Pwajok, Pam Patrick Nyam, Nusrat Husain

**Affiliations:** ^1^ Division of Psychology and Mental Health, The University of Manchester, Manchester, United Kingdom; ^2^ School of Social Science, Humanity and Law (SSSHL), Department of Humanities and Social Sciences, Teesside University, Middlesbrough, United Kingdom; ^3^ Nottingham Trent University, Nottingham, United Kingdom; ^4^ Department of Sociology, Coal City University, Enugu, Nigeria; ^5^ Department of Psychology, University of Jos, Jos, Nigeria; ^6^ Mersey Care National Health Service (NHS) Foundation Trust, Liverpool, United Kingdom

**Keywords:** postnatal depression (PND), British African Caribbean women, cognitive behaviour therapy (CBT), learning through play, cultural adaptation, UK

## Abstract

**Background:**

One in every three women worldwide experiences postnatal depression after childbirth, with long-term negative consequences on their children. The mainstream mental healthcare provision for British mothers of African/Caribbean origin is mostly unsuccessful due to a lack of culturally appropriate care.

**Methods:**

The study adopts a mixed-methods randomised controlled trial (RCT) design. A 12-session (60 minutes each) of online Learning Through Play plus Culturally adapted Cognitive Behaviour Therapy (LTP+CaCBT) intervention was employed for treating postnatal depression in comparison with psychoeducation (PE). Participants aged 19–53 were screened for depression using the Patient Health Questionnaire (PHQ-9). N=130 participants who scored >5 on PHQ-9 were randomised into LTP+CaCBT (n=65) or PE (n=65) groups. N=12 focus groups (LTP+CaCBT, n=6; PE, n=6) and n=15 individual interviews (LTP+CaCBT, n=8; PE, n=7) were conducted, transcribed verbatim and analysed.

**Results:**

Satisfaction with intervention (LTP+CaCBT, 72.9%; PE, 65.2%); retention rates (LTP+CaCBT, 91%; PE, 71%); reduction in postnatal depression was higher in LTP+CaCBT on PHQ-9 Md=1.00 with z= -4.046; compared to PE, Md=1.00 with z= -1.504. Both groups showed reduced levels of anxiety on GAD-7 with no significant difference. Emerging themes from the qualitative findings showed increased positive moods, reduced worries about parenting difficulties and the facilitative role of remote intervention.

**Conclusions:**

LTP+CaCBT intervention is culturally appropriate and acceptable and reduces postnatal depression in British mothers of African/Caribbean origin. A fully powered RCT is recommended to evaluate the clinical and cost-effectiveness of LTP+CaCBT, including the child’s outcomes compared with routine treatment as usual.

**Clinical trial registration:**

www.ClinicalTrials.gov, identifier NCT04820920.

## Introduction

The incidence of maternal disorders such as postnatal depression and anxiety was estimated at almost 80 million cases globally, corresponding to over 800,000 years of life lived with disability (YLDs) in women (1). A recent meta-analysis of 14 studies found a worldwide prevalence of depression and suicide attempts to be 210 per 100,000 during postnatal periods (2). About 47% of women experience severe depression and suicidal thoughts after childbirth, with long-term negative consequences on their children and families (3). British African/Caribbean mothers are 9% more likely to suffer from postnatal depression than their White British counterparts (4). Here, the British African/Caribbean include women from Black British, Black African, Caribbeans, or mixed-raced with African or Caribbean origins.

In addition to high rates of single parenting in the British African/Caribbean population, the women are more likely to be ignored by mental healthcare providers, not followed up on their current or past mental health problems, and not offered any form of treatment for postnatal depression compared with their White counterparts (5). This is partly due to the service-providers’ lack of knowledge around cultural diversity within the context of British African/Caribbean perspectives of the aetiology, diagnosis, and treatment of postnatal depression. Consequently, in the UK, maternal deaths due to mental and physical health complications amongst African/Caribbean women are 41.1 per 100,000 pregnancies compared with 11.1 per 100,000 pregnancies of their White women counterparts (6). Of these deaths, 23% of women who died in the postnatal period suffered from depression and other mental health disorders (7).

Despite these well-documented racial inequalities in maternal health outcomes for African/Caribbean mothers and their children, the quality of care received has increasingly become poorer than other ethnic groups in the UK (8). A few research attempts to explore the maternal health of Black Asian and Minority Ethnic (BAME) communities (9). Thus, the term ‘BAME’ is critiqued for including many ethnic people with different lived experiences and grouping them may be culturally inappropriate (10). For example, African/Caribbean people have historically and still suffered from the legacies of slavery, colonialism, and systemic racial oppression that are unique to them (10). These racial stratifications could be illustrated further with Critical Race Theory (CRT) and Intersectionality Theory. CRT provides the theoretical orientation that underscores racial mental health disparities (11, 12). This helps to understand the systemic process of conceptualising race as a socially construed category. CRT helped not only to understand racial disparities in the mental health of the African/Caribbean population but also instrumental in promoting health equity (12).

Similarly, intersectionality theory further illuminated how different social categorisations, such as Black women, mothers of new-borns, African/Caribbean origin, lower social class and mostly socio-economically disadvantaged. This intersectionality significantly impacts their mental health and life chances (13). By intersecting systemic oppressions leading to racism and poverty and the lack of culturally sensitive care, British African/Caribbean women are predisposed more to postnatal depression compared to their White British counterparts (4). Therefore, British African/Caribbean mothers suffer additional detrimental systemic defects, such as the risk of getting their children forcefully taken away by social services or forced detention under the Mental Health Act (14).

These peculiar mental health challenges are also magnified by the lack of policies, poor funding, and budget-holders’ lack of investment in culturally appropriate services for African/Caribbean communities. Our systematic review of previously used psychological interventions for African/Caribbean mothers in High-Income Countries, including the UK, showed significant limitations of existing interventions (10), which recommended an evidence-based and culturally adapted psychological intervention for postnatal depression. Therefore, the present study’s aim and objective are to examine the cultural appropriateness and preliminary clinical effectiveness of Learning Through Play plus Culturally adapted Cognitive Behaviour Therapy (LTP+CaCBT) compared to psychoeducation for treating postnatal depression in British African/Caribbean women.

## Methods

### Design

The study adopts a randomised controlled design comparing LTP+CaCBT with Psychoeducation (PE). This design is considered an innovative ‘gold standard’ for its methodological rigorousness and is highly recommended for examining psychological interventions (15).

### Ethics

The study received ethical approval from the Nottingham Trent University Ethics Committee.

### Recruitment

The research team leveraged on established networks and distributed printed copies of research flyers to places frequented by British mothers of African/Caribbean origin such as restaurants, community venues/organisations for mother-childcare, churches, and mosques across England. The study was also advertised on BBC radio, which increased the study’s recruitment process. Furthermore, the study was promoted in numerous outlets (e.g., university websites, X (Twitter), Facebook, and LinkedIn).

Participants’ inclusion criteria entailed mothers self-identified as British African/Caribbeans who are experiencing postnatal depression due to childbirth/parenting (postnatal depression is operationally defined as the depression suffered by mothers of children between ages 0-3 years); had a child(ren) between 0-3 years; residents in the UK and are available for the study’s duration and follow-up at 12 weeks end of intervention; 18 years+ and able to give informed consent. The exclusion criteria include mothers who were below 18 years; unable to provide informed consent; and experiencing active suicidal ideation or any other severe disorders. There were no records of participants receiving any additional mental health support or pharmacotherapy at the time of this study.

### Randomisation

Participants who met the study’s inclusion criteria were randomised into LTP+CaCBT or PE interventions using Microsoft Excel. From the list of eligible participants, we randomly generated the value of 0 or 1 using the RANDBETWEEN function in Excel, thereby choosing 0 and 1 as the range. All participants with 0 were assigned to the control (PE) group, and all participants with 1 to the experimental (LTP+CaCBT) group. This randomisation method allowed a single sequence of random assignments in which participants were assigned in a 1:1 ratio (16). Thus, all participants had equal chances of receiving LTP+CaCBT or PE interventions. Although the study’s design was unable to double-masked the community health workers who delivered the interventions due to their involvement in manual co-adaptations and the study design. However, all participants were blinded to whether they were assigned to experimental (LTP+CaCBT) or controlled (PE) groups. Both LTP+CaCBT and PE were named parenting interventions; therefore, participants were blind to the two types of intervention as indicated in the recruitment flyers and consent forms. This approach was beneficial for two reasons: (i) it reduced mental health stigma, and (ii) it helped with participants’ blinding. In addition, assessments were conducted by independent researchers who were not involved in the intervention delivery and were unaware of participants’ assignments to experimental or controlled groups.

### Assessment

A screening assessment for postnatal depression was conducted using a Patient Health Question (PHQ-9– 17), and a Clinical-Interview-Scheduled-Revised (CIS-R – 18) was further administered to confirm the diagnosis of postnatal depression. Thus, participants scoring >5 and above on the PHQ-9 were recruited for baseline assessment and randomised into experimental (LTP+CaCBT) or controlled (PE) groups. Consequently, a score of >5 on PHQ-9 is recommended to indicate postnatal depression requiring intervention (19).

The study’s primary outcome measures examine the cultural appropriateness of the intervention, which was assessed using the Service Satisfaction Scale (20) and the Verona Service Satisfaction Scale (21). The following scales were also used to assess the study’s secondary outcome measures with assessments at baseline and 12-week end of intervention using PHQ-9 (17), Generalised Anxiety Disorder Scale (22) and Oslo Social Support Scale (23).

### Cultural adaptation of LTP+CaCBT

The study employed an ‘Iterative Model of Co-adaptation’ (IMC) (24) for the LTP+CaCBT manual to ensure it is culturally appropriate and suitable for British African/Caribbean women. The IMC process includes the following three stages: Stage 1 Translation/co-adaptation of the manual was conducted in Focus Group Participatory Action Research (FG-PAR) with n=21 participants taking cognisance of context-specific factors such as cultural and religious beliefs, language(s), literacy and preferences (25, under review). Participants include key stakeholders (i.e., women, clinicians, and researchers), including Patients and Public Involvement Events (PPIE) to understand cultural influences such as views about postnatal depression (idioms of distress and its perceived causes), language and religious implications, and the type and level of support needed for virtual or in-person intervention. The outcomes and recommendations from the FG-PAR and PPIE were implemented into the manual and reverted accordingly. Stage 2 Manual testing including (i) Stage 2a: An ‘exploratory’ manual testing in a feasibility trial with a small cohort of n=26 participants was conducted (24). (ii) Stage 2b: A ‘confirmatory’ testing of the manual was further conducted in the present study to validate the cultural appropriateness and suitability of LTP+CaCBT. This helped identify potential trade-offs, ethical dilemmas, barriers, and mitigations for future scale up. Stage 3 In future scale-up trials, a process evaluation would be embedded to monitor the LTP+CaCBT manual’s continued suitability for the target population. All relevant feedback (i.e., feasibility and exploratory trials, conferences, seminars, and workshops) are being undertaken to inform the manual’s clinical value, practical utility and how it could be integrated into local care systems.

### Interventions

**Figure 1:**
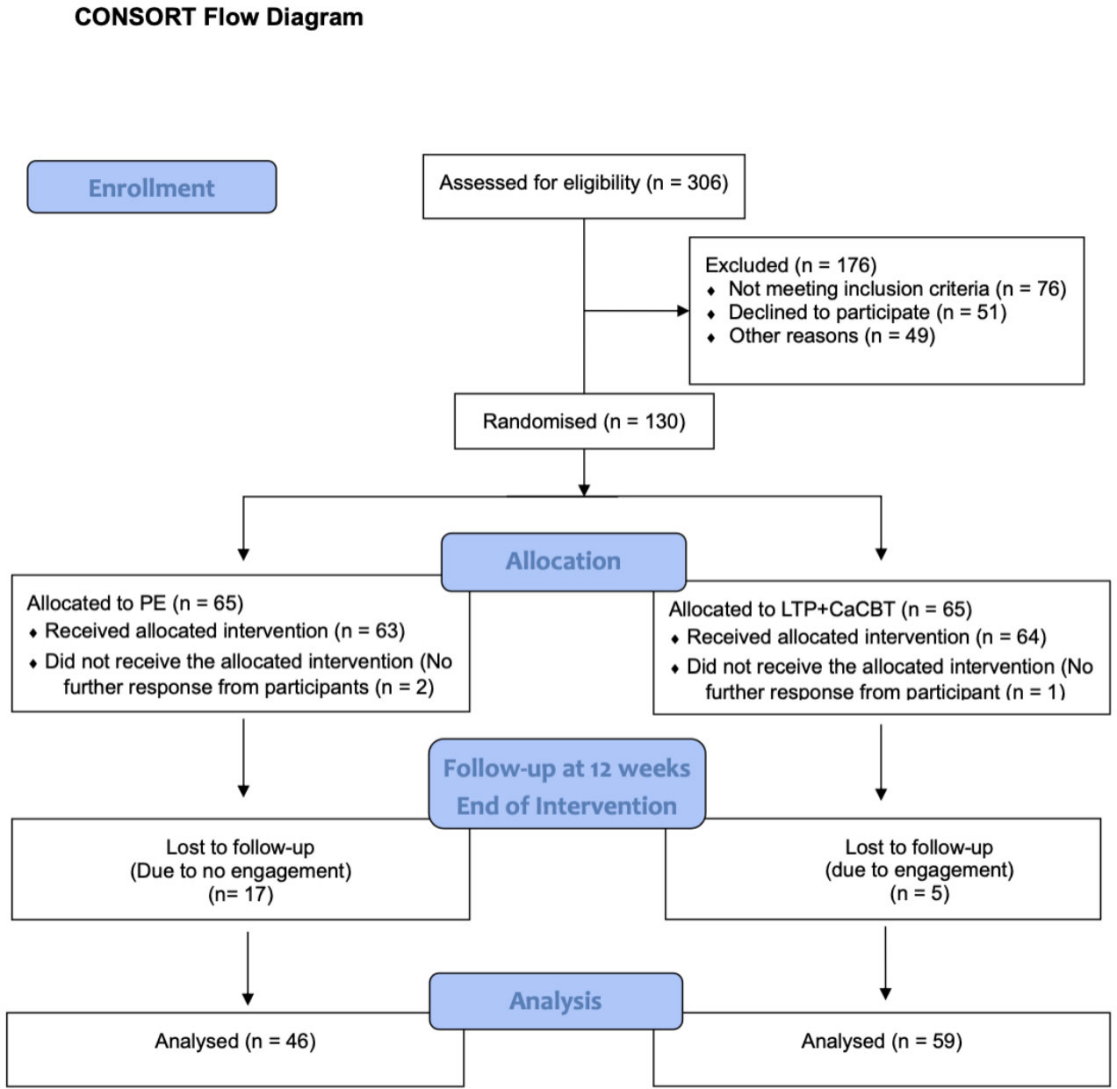
CONSORT Flow Diagram showing enrolment, randomisation, and inclusion of participants in the data analysis.

N=130 eligible participants were recruited (see **Figure 1**) and randomly assigned each to one of two groups: Group 1: Experimental n=65 received online (via MS Teams) ‘LTP+CaCBT’, consisting of 12-group sessions lasting approximately 60 minutes per session every week with 10-12 mother-child pairs per sub-group. Group 2: Controlled n=65 received the online (via MS Teams) PE, consisting of 12-group sessions lasting approximately 60 minutes per session every week with 10-12 mother-child pairs per sub-group. The 12 sessions are the standard recommended duration for brief psychological interventions (26). Because LTP+CaCBT and PE are manualised and the gender-sensitive nature of the interventions, sessions were delivered by trained female Community Health Workers (CHWs) supervised by the Principal Investigator every week. To avoid contamination, dedicated CHWs each facilitated LTP+CaCBT or PE sessions.

### Community health workers training

The study’s CHWs were postgraduate-level non-mental health experts who received (a) three days (24 hours) of extensive trainers’ training on LTP+CaCBT and (b) four days (32 hours) of Global Mental Health research intervention training. In addition, the CHWs were involved in PPI events and LTP+CaCBT manual adaptation. CHWs also received a 1-day (8 hours) Theory of Change training workshop. The use of CHWs complements the World Health Organisation (27) recommendations of task-shifting strategies to use non-mental health professionals to tackle shortages of [culturally knowledgeable] mental health workforce.

### Data analyses

#### Quantitative analysis and outcome measures

*N =* 130 participants were recruited for the study, and data were analysed to determine the study’s primary outcome measures of LTP+CaCBT intervention’s cultural appropriateness and acceptability using participants’ satisfaction with the intervention scales (20, 21). For secondary outcome measures, an inferential statistic using the Wilcoxon sign rank test of analysis was conducted to determine the intervention’s preliminary clinical effectiveness in treating postnatal depression and anxiety (17, 22). This non-parametric statistical model was most appropriate due to skewness and the inability to validate data distribution across experimental and controlled groups (28).

#### Qualitative analysis and interpretative phenomenological analysis

N = 12 focus groups (LTP+CaCBT, n = 6; PE, n = 6) and n = 15 individual interviews (LTP+CaCBT, n = 8; PE, n = 7) were conducted online via MS Teams. Qualitative data was collected until saturation was reached, at which point no new findings were identified. All interviews were recorded, transcribed verbatim and analysed using an interpretative phenomenological analysis (IPA), the most appropriate qualitative method for examining participants’ lived experiences of their postnatal depression and interventions. All participants’ identifiable information, such as their names in individual interviews and focus group discussions, were pseudonymised to retain the IPA ideographic nuances in the dataset. The result section has used relevant data verbatim from the transcripts to support emerging themes. The data analysis, analytical commentaries and the interpretations of findings were underpinned by IPA theoretical features, including phenomenology, single- and double-hermeneutics and idiographics (29, 30).

The study’s qualitative arm helped capture rich datasets associated with participants’ implicit nuances of their experience of the intervention and postnatal depression from their defined perspectives in ways that could not have been possible using structured psychometric tools or questionnaires (31).

## Results

### Quantitative: primary outcome measure

Based on **Table 1** above, we observed high retention rates across both groups. As shown, those assigned to the experimental group indicated a higher level of participation at 82% compared to the controlled group PE, with an attendance rate of 74%. Part of the high retention could be attributed to the LTP+CaCBT, which is culturally adapted and draws on content including scenarios the participants could relate to and the interactive nature of the sessions.

**Table 1:** Participants’ attendance for LTP+CACBT and PE.

Sessions	LTP+CACBT *N=*65		PE *N=*65	
	*n*	%	*n*	%
1	54	83	39	60
2	51	78	41	63
3	54	83	44	68
4	52	80	47	72
5	58	89	48	74
6	54	83	49	75
7	47	72	53	82
8	57	88	47	72
9	57	88	50	77
10	54	83	52	80
11	48	74	52	80
12	51	78	53	82
% ÷ 12 sessions_ = _Total		82		74

Key: N = total number of participants randomised to each arm, n = total number of participants who attended each session. The percentage comprises those who attended out of 65 participants in each arm.

Overall, the experimental group denotes a higher level of satisfaction than the control group (**Table 2**). Even in terms of the perceived effectiveness of the intervention, those in the experimental group reported higher effectiveness. This finding suggests that the LTP+CaCBT appears more acceptable when compared to the PE in controlled intervention. In the customer service satisfaction scale, n=6 participants did not fill this specific survey for the experimental group (LTP+CaCBT) whilst n=3 participants did not fill this ambit for the controlled group (PE). This is why the percentage for each statement did not tally at 100%.

**Table 2:** Intervention satisfaction.

S/N	Survey: For the following questionnaire, please provide responses according to your experience of the service provided.	LTP+CaCBT *n* = 59 %					PE *n* = 46 %				
		0	1	2	3	4	0	1	2	3	4
1.	Acceptability 0 = not at all useful; 1 = Slightly useful; 2 = Moderately useful; 3 = Very useful; 4 = Extremely useful.			5.1	35.6	49.2	2.2	6.5	4.3	50.0	30.4
2.	Effectiveness 0 = not at all useful; 1 = Slightly effective; 2 = Moderately effective; 3 = Very effective; 4 = Extremely effective.			10.2	39.0	40.7	2.2	8.7	6.5	45.7	30.4
3.	Intrusiveness 0 = Extremely uncomfortable; 1 = Somewhat uncomfortable; 2 = Neither comfortable nor uncomfortable; 3 = Somewhat comfortable; 4 = Extremely comfortable.			10.2	42.4	37.3		10.9	2.2	39.1	41.3
4.	Quality 0 = Extremely bad; 1 = Somewhat bad; 2 = Neither good nor bad; 3 = Somewhat good; 4 = Extremely good.			3.4	28.8	57.6			6.5	32.6	54.3
5.	Satisfaction 0 = Extremely dissatisfied; 1 = Somewhat dissatisfied; 2 = Neither satisfied nor dissatisfied; 3= Somewhat satisfied; 4 = Extremely satisfied			1.7	25.4	62.7		8.7	4.3	26.1	54.3
6.	Recommend to others 0 = Definitely not; 1 = Probably not; 2 = Might or might not; 3= Probably yes; 4 = Definitely yes.				15.3	74.6			14.0	19.3	66.7

Similarly, the experimental group received a higher level of satisfaction when compared to the controlled group (**Table 3**). For example, 72.9% of participants expressed the ‘amount of help received’ as extremely good in the experimental group compared with 65.2% of the controlled group. Again, the retention level for the experimental group at 59 (91%) by the end of the intervention also appears higher than that of the controlled group with 46 (71%) participants by the end of the intervention. In terms of improvement of service in helping patients improve their capacity to look after themselves, the experimental group also reported higher scores at 45.8% (extremely effective) and 39.0% (very effective) when compared to the controlled group, which had a score of 28.3% (extremely effective) and 39.1% (very effective) with some 4.3% indicating not effective at all.

**Table 3:** Verona service satisfaction scale.

S/N	Survey: For the following questionnaire please base your answers on your experience of the service you were provided.	LTP+CaCBT *n* =59 %					PE *n* = 46 %				
		0	1	2	3	4	0	1	2	3	4
1.	Amount of help received. 0 = Extremely bad; 1 = Somewhat bad; 2 = Neither good nor bad; 3=Somewhat good; 4= Extremely good.	00	00	3.4	23.7	72.9		6.5	4.3	23.9	65.2
2.	Kind of services 0 = Not at all useful; 1 = Slightly useful; 2 = Moderately useful; 3 = Very useful; 4= Extremely useful.	00	00	5.1	52.5	42.4		4.3	26.1	37.0	32.6
3.	Overall Satisfaction 0 = Extremely dissatisfied; 1= Somewhat dissatisfied; 2 = Neither satisfied nor dissatisfied; 3 = Somewhat satisfied; 4 = Extremely satisfied.	00	00	1.7	32.2	66.1			8.7	23.9	67.4
4.	Explanation of specific procedures and approaches used. 0 = Never; 1 = Sometimes; 2 = About half the time; 3 = Most of the time; 4 = Always.	00	00	3.4	37.3	59.3		8.7	4.3	30.4	56.5
5.	Information on diagnosis and prognosis 0 = Extremely unclear; 1 = Somewhat unclear; 2= Neither clear nor unclear; 3 = Somewhat clear; 4 = Extremely clear	00	00	1.7	40.7	57.6		6.5	4.3	37.0	52.2
6.	Publicity or information on mental health services which are offered. 0= A little; 1 = A little; 2 = A moderate amount; 3= A great deal; 4 = Extremely clear.	00	1.7	10.2	39.0	49.2	2.2	10.9	21.7	43.5	21.7
7.	Effectiveness of the service in attaining well-being and preventing relapses 0 = Not effective at all; 1 = Slightly effective; 2 = Moderately effective; 3 = Very effective; 4 = Extremely effective.	00	00	8.5	55.9	35.6	4.3	6.5	8.7	58.7	21.7
8.	Effectiveness in the service in helping patients deal with problems. 0 = Not effective at all; 1 = Slightly effective; 2 = Moderately effective; 3 = Very effective; 4 = Extremely effective.	00	00	3.4	55.9	40.7	2.2	10.9	15.2	41.3	30.4
9.	Effectiveness of the service in helping patients to improve knowledge and understanding of his/her problems. 0 = Not effective at all; 1 = Slightly effective; 2 = Moderately effective; 3 = Very effective; 4 = Extremely effective.	00	00	5.1	59.3	35.6	2.2	10.9	13.0	47.8	26.1
10.	Effectiveness of the service to help patients relieve symptoms. 0 = Not effective at all; 1 = Slightly effective; 2 = Moderately effective; 3 = Very effective; 4 = Extremely effective.	00	00	11.9	54.2	33.9	4.3	6.5	19.6	50.0	19.6
11.	Effectiveness of the service in improving the relationship between patient and relative 0 = Not effective at all; 1 = Slightly effective; 2 = Moderately effective; 3 = Very effective; 4 = Extremely effective.	00	1.7	10.2	47.5	40.7	4.3	8.7	21.7	45.7	19.6
12	Effectiveness of the service in helping the patient improve capacity to look after him/herself. 0 = Not effective at all; 1 = Slightly effective; 2 = Moderately effective; 3 = Very effective; 4 = Extremely effective.	00	00	15.3	39.0	45.8	4.3	8.7	19.6	39.1	28.3
13.	Effectiveness of the service in helping patients establish good relationships outside the family environment. 0 = Not effective at all; 1 = Slightly effective; 2 = Moderately effective; 3 = Very effective; 4 = Extremely effective.	00	00	11.9	44.1	44.1	4.3	8.7	8.7	52.2	26.1
14.	Effectiveness of the service in helping patients improve abilities to work. 0 = Not effective at all; 1 = Slightly effective; 2 = Moderately effective; 3 = Very effective; 4 = Extremely effective.	00	1.7	11.9	49.2	37.3	2.2	13.0	21.7	37.0	26.1

### Quantitative: secondary outcome measure

The PHQ-9 have a total score of 27, with 1 denoting a score of between 0 and 4, ranked at no depression. Two denotes a score of between 5 and 9, ranked at mild depression, whilst three represent sores within the range of 10 and 16, indicating moderate depression, and four represents scores between 15 and 19, interpreted to mean moderately severe depression. Finally, five encompasses scores between 20-27, which means severe depression. See **Table 4** below, showing medians and category scores across time points for experimental (LTP+CaCBT) at baseline and End of Intervention (EoI) compared to the control group (PE).

**Table 4:** Scores across time points at baseline and end of intervention.

Scales	Baseline (EG) *Md*	EoI *Md*	*z*	*p*	Baseline (CG) *Md*	EoI *Md*	*z*	*p*
			Baseline * EoI				Baseline * EoI	
PHQ-9	2.00	1.00	-4.046	.000	2.00	1.00	-1.504	.132
GAD-7	1.00	1.00	-4.062	.000	1.00	1.00	-.261	.794
OSSS	1.00	2.00	-5.929	.000	1.00	2.00	-4.185	.000

PHQ-9, Patient Health Questionnaire; GAD-7, Generalised Anxiety Disorder; OSSS, Oslo Social Support Scale; EoI, End of Intervention; EG, Experimental Group; CG, Controlled Group.

**Table 4** above highlights the results of PHQ-9 at baseline for the experimental group, which denotes some reduction in depression from *Md*=2.00 at baseline to *Md*=1.00 with *z=*-4.046 at the end of the intervention. This improvement shows that LTP+CaCBT positively impacts the participants’ moods. The PHQ-9 results are based on all 65 participants’ completion at baseline and 59 at the end of the intervention. The GAD-7 data suggest that 63 participants completed this specific survey at baseline and 59 at the end of the intervention. In line with the analysis, it was found that the score appears stable for the GAD-7 with not much difference between *Md*=1.00 at baseline and *Md*=1.00 at the end of intervention with *z=-*4.062. For the OSSS, a similar number of participants (e.g., 63) completed this specific survey at baseline, whilst 59 completed it at the end of the intervention. The result highlights moderate social support improvement from *Md*=1.00 at baseline to *Md*=2.00) with *z=-5.929.* This denotes the overall positive benefit of the LTP+CaCBT intervention.

For the controlled group, which was based on the Psychoeducation (PE) intervention, the results suggest that the PHQ-9 at baseline showed some reduction in depression from *Md*=2.00 at baseline to *Md*=1.00 with *z*=-1.504 at the end of the intervention. While there were some improvements, the experimental group reported a higher z=-4.046 based on positive rank compared to the controlled group at *z*=-1.504, thus denoting more improvement than the controlled group. This could be due to the higher retention rate for the experimental group, as the controlled group had 65 participants who filled out the baseline survey for the PHQ-9 and n=46 participants completing the survey at the end of the intervention. The GAD-7 data indicated completion by n=65 participants at baseline and n=46 participants at the end of the intervention. The analysis found that the score appears stable for the GAD-7 with *Md*=1.00 at baseline and *Md*=1.00 at the end of intervention with *z*=-.261.

Similarly, for the OSSS, data indicated completion by 65 participants at baseline and n=46 at the end of the intervention. The result highlights some improvement in social support from *Md*=1.00 at baseline to *Md*=2.00) with *z*=-4.185. Again, compared to the experimental group, the experimental group reported a higher z-value at z=-5.929, showing more improvement in social support. The higher value may be due to the higher retention rate compared to the psychoeducation group.

### Qualitative findings

#### Theme 1: positive experiences of the intervention led to increased confidence in parenting

Participants from the experimental group recount their positive experiences and satisfaction with the intervention. One of the participants said:

*What I have learned throughout this parenting training[LTP+CaCBT, is essential because I learned a lot about how to know the stages of my child’s development when she is growing up and then the things that I will be seeing in her and that I might be thinking that I cannot deal with it, but throughout the training, I got a good experience of how and what to do when it comes to that* (Benita, a 30-year-old mum of an 18-month-old baby, EG).

Similarly, another mother commented that:

*[The LTP+CaCBT] was good. At the time, it made me think of my parenting skills and parenting techniques, which were really good (Chiumbo, 38-year-old mom of 24-month*-old baby, EG).

The above extracts depicted Benita and Chiumbo’s experiences and the role of the LTP+CaCBT in shaping their parenting skills as enormously beneficial. Content incorporating hypothetical scenarios and dealing with parenting issues were very helpful in managing their child(ren) and enhancing their parenting skills. However, the controlled group acknowledges the usefulness of the intervention with some reservations. For example, one participant says:

*I appreciate what has been done, but I would rather want to prepare ahead of time. I will think that if we have the topic ahead of time before we start this program. We will know that we’re going to prepare something. I don’t have a clue what the topic is for the next section (Diolla, 34-year-old mom of 12-month*-old baby, CG).

Diolla found the session educative. However, it is recommended that it will be beneficial to notify all participants of what each session of intervention activities will be covered in advance.

#### Theme 2: usefulness of real-life parenting scenarios and the reshaping of parenting cultural norms

Another dominant theme in the dataset was the perceived view that the training material was highly educative and helpful. Participants expressed satisfaction with the scenarios, educative videos embedded in the training material, and the overall content. For example, one mother with a young child aged two at the time of the intervention commented that:

*Yes, the scenarios used and how we dealt with them were going on in parenting. It was a fact story that was going on. I even experienced some of the scenarios the lecturer [Community Health Worker delivering the LTP+CaCBT intervention] brought, which was very useful. Yes, I find it very useful and relevant* (Gina, 28-year-old mum of 24 months-old baby, EG).

Another mother, whilst reflecting on the training materials, said that:

*I think it was very impactful. [ … ] It was just interesting to learn new facts to put off some old parenting habits. It was just very, very informative too, indeed; I must say it was very eye-opening too. I found it very useful. It was really nice and I was able to learn a lot from it* (Fatima, 42-years-old mum of 36-months-old baby, EG).

*I think it’s more about the myth busters, so when I was growing up, we were told that things are done a certain way and in some traditions and norms* (Kaleisha, 26-year-old mum of a 9-month-old baby, EG).

Gina, Fatima and Kaleisha felt the intervention content was informative, collectively denoting the educative nature of the LTP+CaCBT training materials. Regarding the controlled group, one participant said:

*A particular case study that was used about a mum who probably didn’t have the best support system, I had that kind of experience too when I had my first child. So yeah, I could definitely relate* (Orla, 45-year-old mum of a 30-month-old baby, CG).

Orla’s reflection depicted how the programme’s contents and scenarios were perceived as helpful to her lived experience.

#### Theme 3: facilitative role of remote parenting intervention

Remote intervention increased parenting efficiency. Commenting on this, one participant from the EG recounts her positive learning from the online intervention as follows:

*My husband actually joins the online training, so he will be listening in the background and taking notes, so we have learned* that for the next child. The mistakes that we *made before joining this group session, we know how to move forward with it, especially when having a baby during a pandemic, I did not have access to all the resources I needed. This has been very- it’s been extremely useful* (Kaleisha, 26-year-old mum of a 9-month-old baby, EG).

Kaleisha narrated her lived experience with the usefulness of the intervention as it relates to parenting skill acquisition. The remote delivery primarily facilitated the husband’s ability to attend the intervention. Another participant also noted:

*To be honest, this online training should be made available to all mums in the UK* (Sarah, 29-year-old mum of 12-months-old baby, EG).

Another mum also commented on the usefulness of the remote intervention:

*The little I’ve learnt in this online training now, being a mum, I would say it is one of the most painful situations where I felt like, as a Black race, we are not being catered for in terms of, you know, no support, no guidance and having that community of Black people to have that kind of relationship, especially for new mums who are struggling with parenting, who are probably single mums who are mothers, who have no support, no family…* (Olusola is a 28-year-old mum of a 12-month-old baby, CG).

The above extract shows Olusola’s experiences as a Black woman, illustrating the potential struggles of other women of British African/Caribbean origin.

#### Theme 4: reduced postnatal distress and increased positive moods

Participants also expressed how the intervention has significantly reduced postnatal depression, anxiety and other parenting issues relating to emotional distress. In the experimental group, whilst commenting on the appropriateness of the information during the intervention and its usefulness, one participant said:

*The cultural materials and the information that we were able to compare were very useful because if I’m able to communicate better with my child, it helps me develop a better relationship with him, and the ripple effect of that is my mental health is improved because I enjoy being a mother* (Kiyana, 32 years-old with 36-months-old baby, EG).

As the extract indicates, Kiyana highlights how her mental health improves due to engaging in culturally relevant parenting that increases mother-child bonding, and attachment. As one participant from the controlled group said:

*For a woman to give birth instead of being the happiest person for a little while, but postnatal depression sets in. So, I learned some things I never knew about before. So, it was a very good, very good experience. Because now we have so many people from African, Caribbean countries coming over [to the UK], and they don’t know their way around emotional problems of parenting. The dos and don’ts of this country, so they fall victim to social work. The parenting awareness of this program will help them* (Nala, 37-years-old mum of 18-months-old baby, CG).

The preceding extract highlights a lack of awareness of the norms and values embedded in the UK’s parenting culture, which some parents from the African/Caribbean communities may be unaware of. The consistent hesitance in the above extract also suggests high levels of uncertainty about parenting within the UK context.

## Discussion

This study examines the acceptability and cultural appropriateness of LTP+CaCBT intervention for treating postnatal depression in British mothers of African/Caribbean origin. Quantitative findings of primary outcome measures showed higher satisfaction in the LTP+CaCBT (experimental group) compared to the psychoeducation (controlled group). Thus, the LTP+CaCBT group reported higher levels of participants’ retention, engagement, and overall intervention effectiveness. These findings imply that the LTP+CaCBT is more acceptable and culturally appropriate for treating postnatal depression when compared to Psychoeducation (PE) in British African/Caribbean mothers. These differences could be attributed to the well-crafted nature of the LTP+CaCBT intervention’s activities that resonate with the participants’ cultural values, stimulating sustainable engagement and treatment outcomes.

The value implication of cultural appropriateness in maternal mental health care is consistent in African/Caribbean mothers (24). For example, in another study in Kenya, LTP+CaCBT was found to be culturally appropriate and acceptable for treating postnatal depression in rural, low-income and socio-economically disadvantaged women (32). However, it is worth mentioning the cautionary observations made in Jidong et al. (24) and Notiar et al. (32) studies which were both feasibility trials with primary outcome measures testing the appropriateness and acceptability of the LTP+CaCBT intervention with small sample sizes of the former n=26 (double arm) and the later n=19 (single arm). Therefore, they were not powered to evaluate the clinical and cost-effectiveness of the LTP+CaCBT intervention. However, despite the small sample sizes and lack of statistical power, the secondary outcome measures in both studies showed a consistent reduction in depression and anxiety scores with increased social support networks at post-intervention measures (24, 32).

Other studies have adapted similar psychosocial interventions for depressed mothers in other cultures. For example, in two randomised controlled trials, Husain et al. (33) examined the treatment of maternal depression in urban slums of Karachi, Pakistan, using an integrated maternal psychological and early child development intervention; and Husain et al. (34) further evaluated the efficacy of learning through play plus (LTP+) intervention to reduce maternal depression in women with malnourished children in Pakistan. Findings from Husain et al. (33, 34 showed that mother-child attachment and play activities improved maternal mood and reduced depression – the mother’s quality of life and the child’s well-being significantly improved. So far, the present study and discussed studies have shown significant relevance of cultural adaptations in maternal mental health in collectivist cultures of African/Caribbean and Pakistani communities. Thus, collectivist cultural values and practices were integrated into psychological interventions, typical features of African, Caribbean and South Asian communities in the diaspora and their indigenous countries.

Furthermore, the secondary outcome measures showed higher reductions in postnatal depression and anxiety and increased social support networks in the LTP+CaCBT group compared to the psychoeducation group. The study’s qualitative findings further depicted that the LTP+CaCBT intervention provided the mothers with stimulating parenting knowledge about early child development underpinned by the theory of attachment (35) and integration of healthy thinking patterns underpinned by the theory of cognitivism (36). For example, the cognitive behaviour therapy component of the intervention uses active listening techniques, changing negative thinking, and guided discovery such as the Socratic questioning style to gently probe for cultural beliefs on mental health and stimulate alternative positive thinking. These theoretical lenses embedded in the LTP+CaCBT process could predict the intervention’s sustainability. In addition, the remote provision of the LTP+CaCBT intervention increased participation and retention (37, 38).

## Limitations

The intervention in the present study was delivered remotely due to ethical considerations during the COVID-19 pandemic. Therefore, the authors recommended future trials to be conducted face-to-face and identify potential nuances associated with in-person delivery. The present study was not statistically powered to detect the clinical and cost-effectiveness of LTP+CaCBT intervention for treating postnatal depression due to limited funds and resources at the time of this study. As such, future studies should consider conducting fully powered trials comparing LTP+CaCBT with the NHS routine Treatment As Usual (TAU). Another limitation is the non-measuring clinical implication of postnatal depression on the children of depressed women. Thus, children’s outcomes were not captured in the present study. Future trials should consider measuring mother-child attachment and overall child outcomes. More so, the task-shifting model is criticised for the quality of the training and the time to train low-intensity therapists. However, the present study ensured that CHWs had clearly defined roles with appropriate skill training and incentives to deliver the intervention (39). Although there seems to be some contentious nature of using IPA in focus group settings; however, the value addition of the focus group discussion was instrumental to stimulating nuance conversions that facilitated collective and reflexive live experiences of postnatal depression and the intervention received.

## Study’s contribution and clinical implication

Postnatal depression is a significant public health concern leading to global intergenerational mental health disease burden in women, children, and their families. This study’s intervention has contributed to mitigating the disease burden of postnatal depression and further reducing potential child-related disorders in the cohort of British African/Caribbean mother-child pair beneficiaries.

The LTP+CaCBT intervention also contributed towards the United Nations (40) Sustainable Development Goals (SDGs), which include: SDG-3 – which provided evidence-based insights into treating postnatal depression in British African/Caribbean mothers who have no access to culturally relevant care; SDG-4/10 – addressed inequality and inequity in the current state of postnatal mental health provision; SDG-5 – addresses postnatal depression, which is a gender-based disease that disadvantages British African/Caribbean women; and SDG-17 – provides the researchers with a new partnership of teamwork, laying the foundation transferability into other populations and contexts such as African and Caribbean countries.

## Conclusion

Postnatal depression is likely to be prolonged in women due to childbirth and maternal distress with potential intergenerational consequences. The mainstream mental healthcare provision for British mothers of African/Caribbean origin is mostly unsuccessful due to a lack of culturally appropriate care. However, our LTP+CaCBT has shown to be acceptable and culturally appropriate and reduces depression in British mothers of African/Caribbean origin. Participating mothers showed increased positive moods and reduced worries about parenting difficulties or maternal distress underpinned by sustainable mental health self-care strategies embedded in LTP+CaCBT. The facilitative role of the remote intervention increases participation and retention. Finally, a fully powered RCT is recommended to evaluate the clinical and cost-effectiveness of LTP+CaCBT, including children’s outcomes compared with the NHS routine treatment as usual.

## Data availability statement

The datasets presented in this article are not readily available because all relevant data are presented in this paper, and due to ethical considerations, no further data will be available to the public. Requests to access the datasets should be directed to the corresponding author.

## Ethics statement

The studies involving humans were approved by Nottingham Trent Research Ethics Committee. The studies were conducted in accordance with the local legislation and institutional requirements. The participants provided their written informed consent to participate in this study.

## Author contributions

DJ: Conceptualization, Data curation, Formal analysis, Funding acquisition, Investigation, Methodology, Project administration, Resources, Writing – original draft, Writing – review & editing. TI: Conceptualization, Investigation, Methodology, Project administration, Validation, Writing – review & editing. MM: Data curation, Investigation, Writing – review & editing. CF: Data curation, Investigation, Writing – review & editing. SM: Data curation, Investigation, Writing – review & editing. JJ: Data curation, Investigation, Writing – review & editing. JP: Conceptualization, Investigation, Writing – review & editing. PN: Conceptualization, Methodology, Writing – review & editing. NH: Conceptualization, Methodology, Writing – review & editing.
